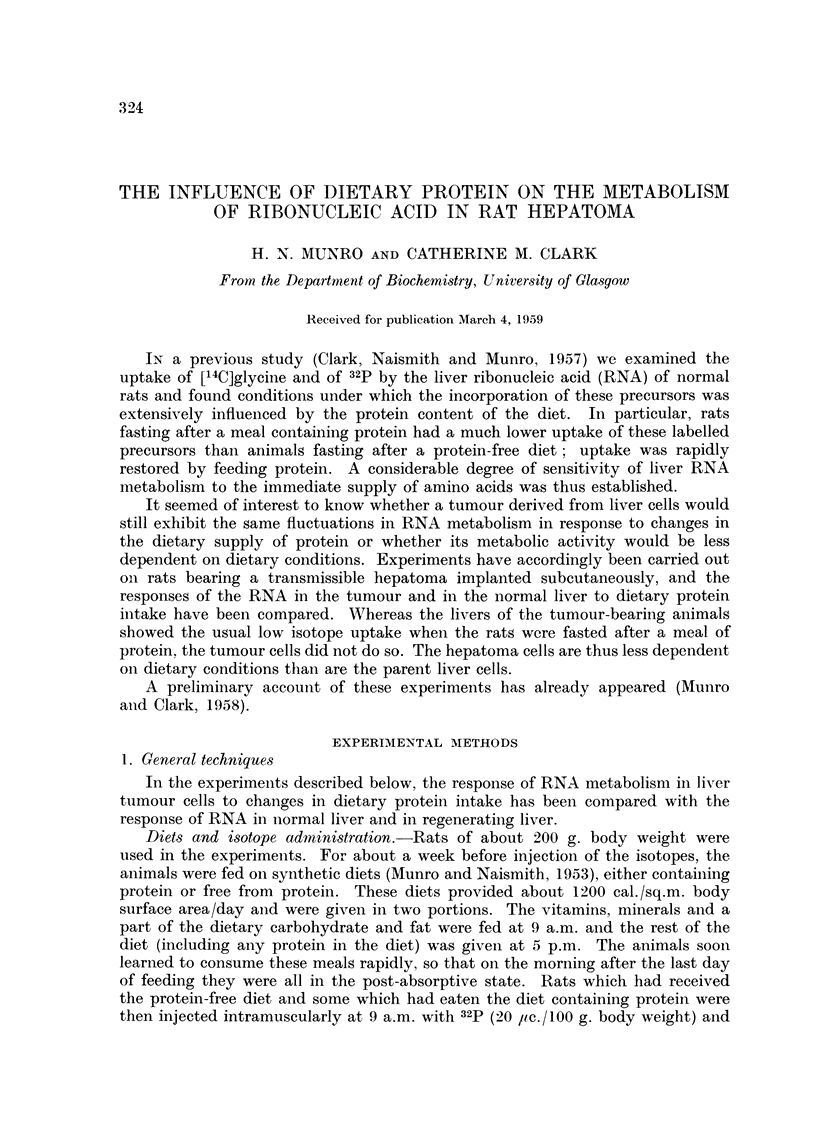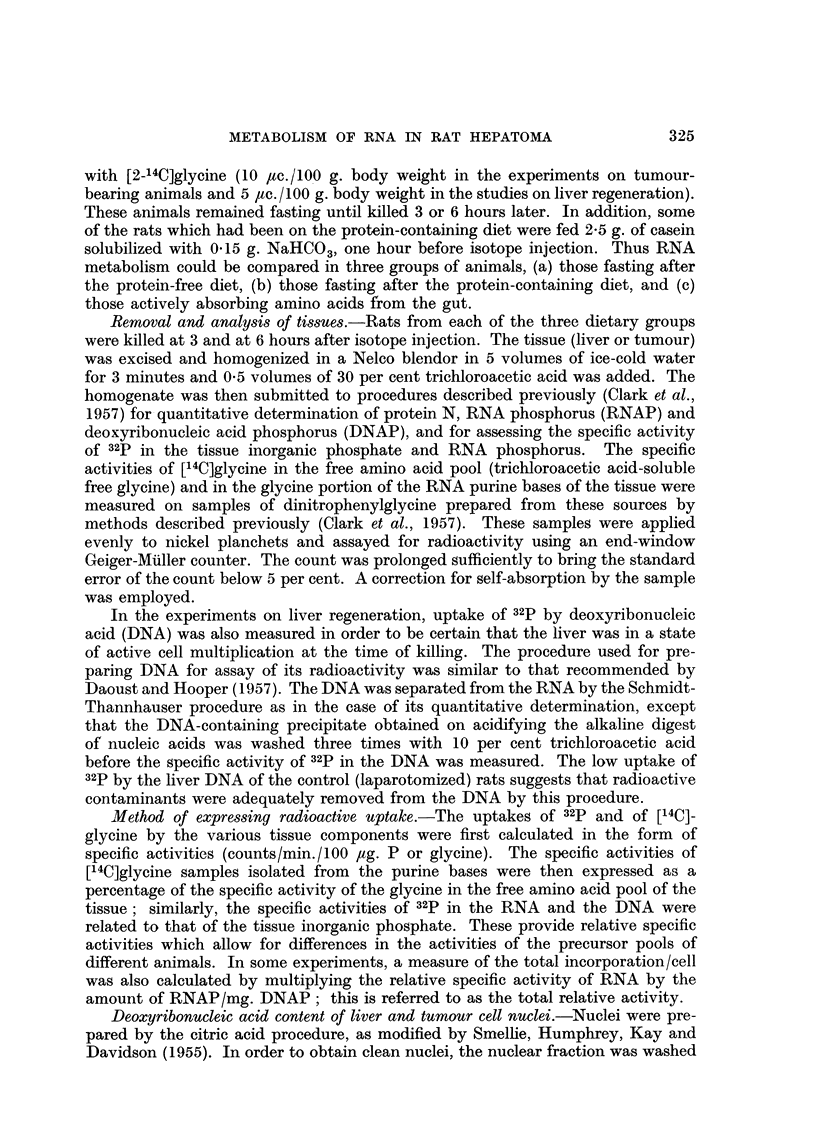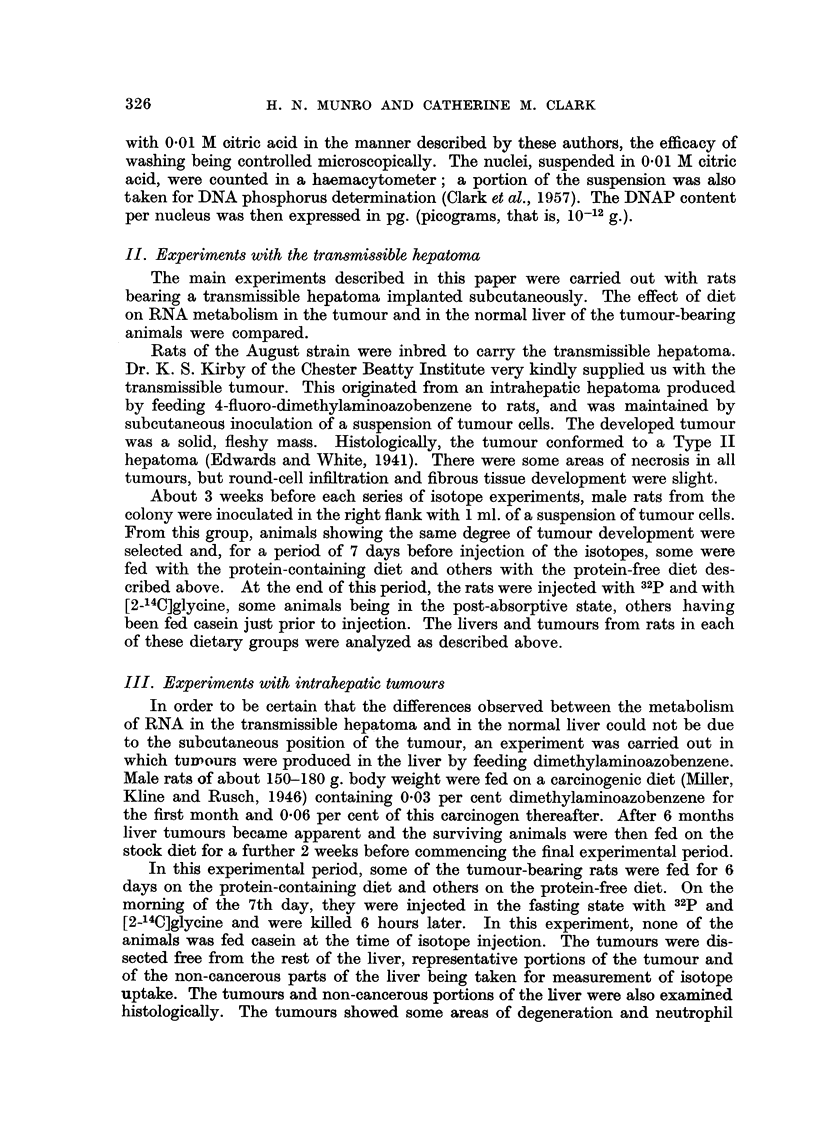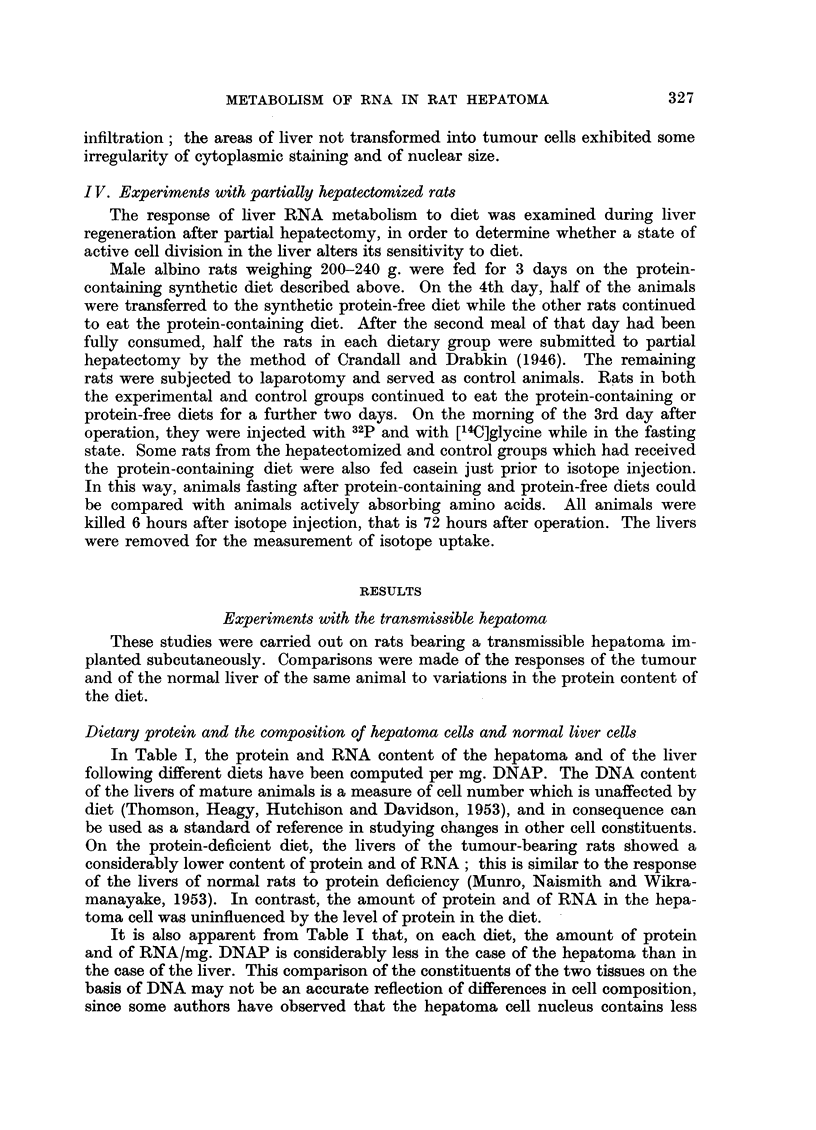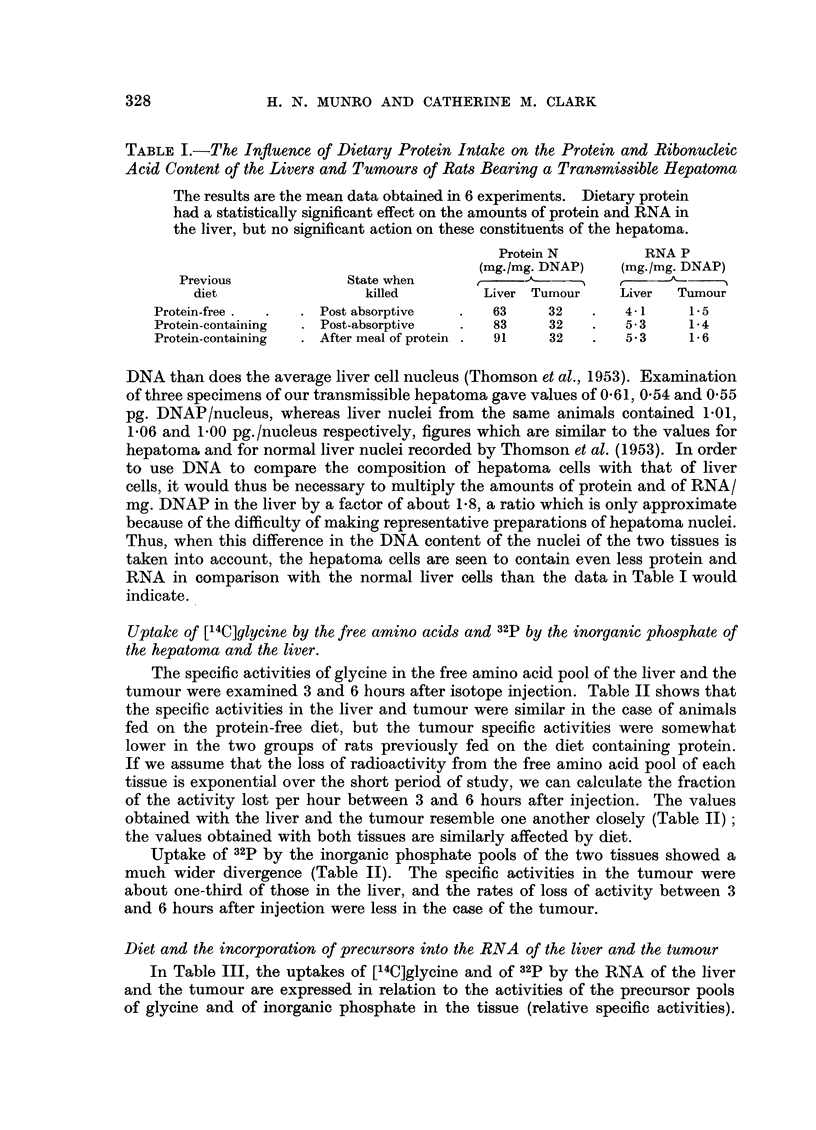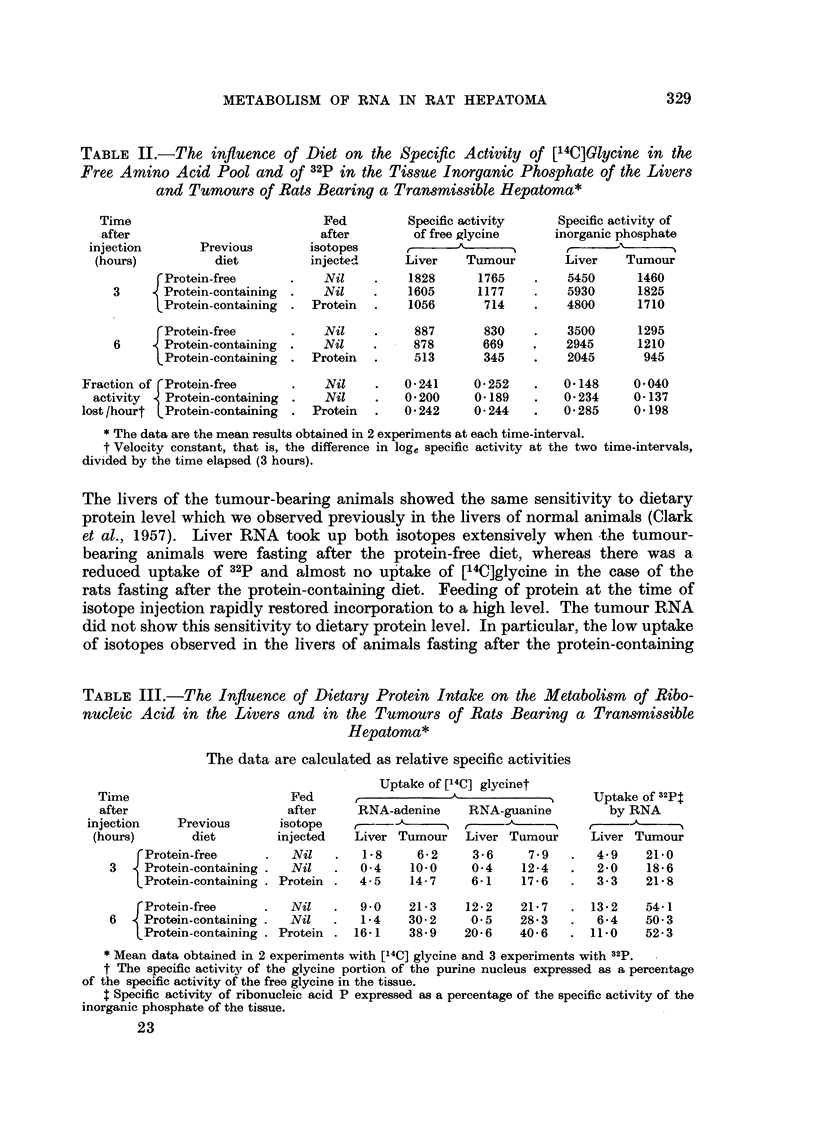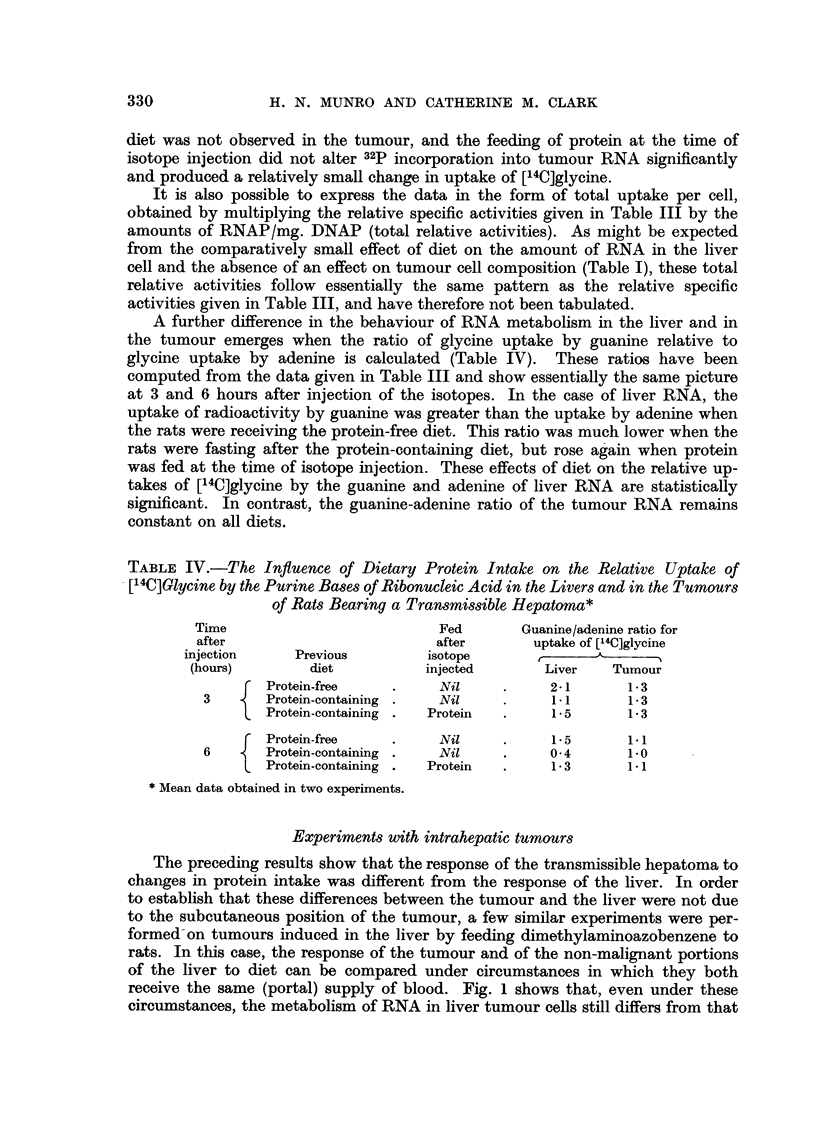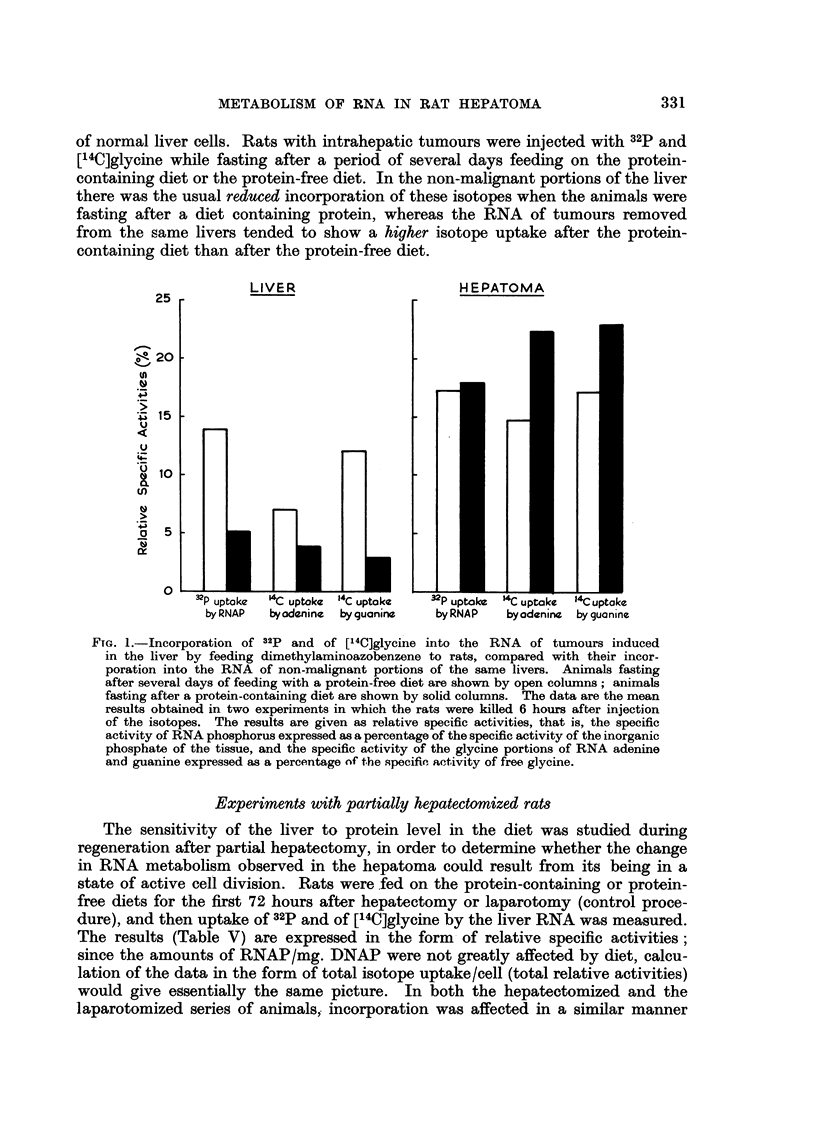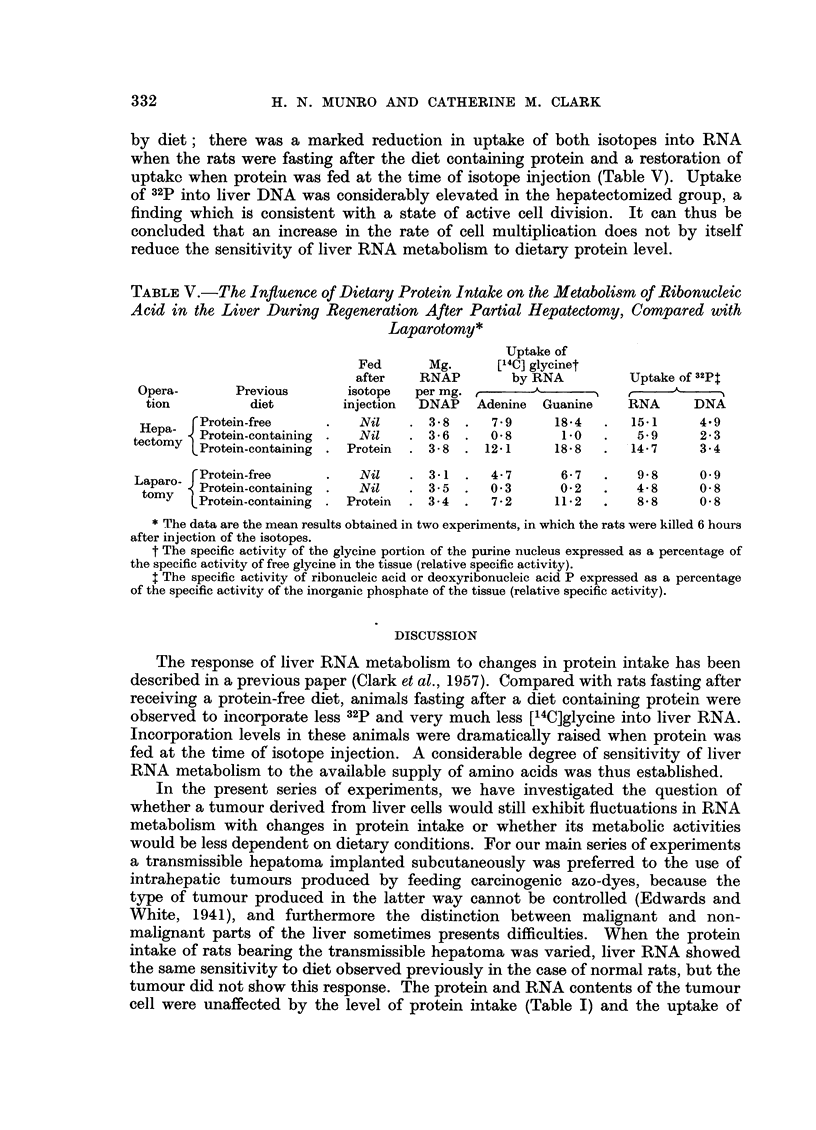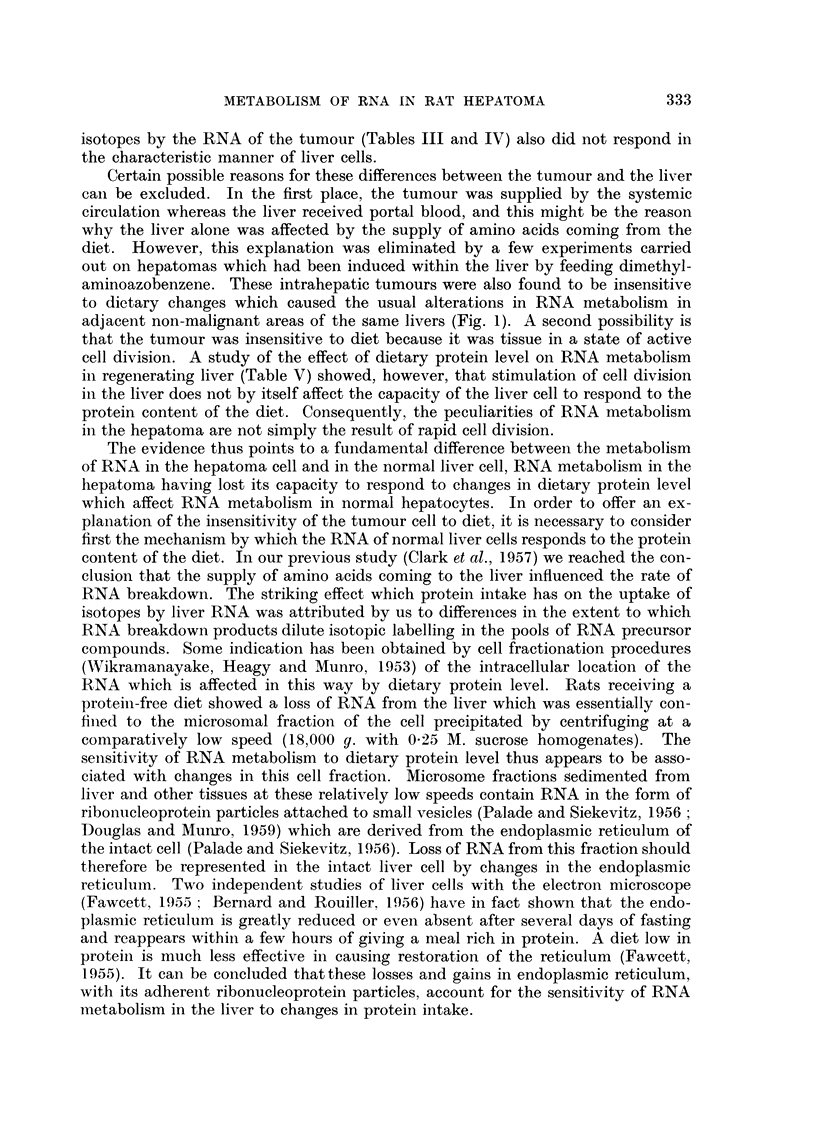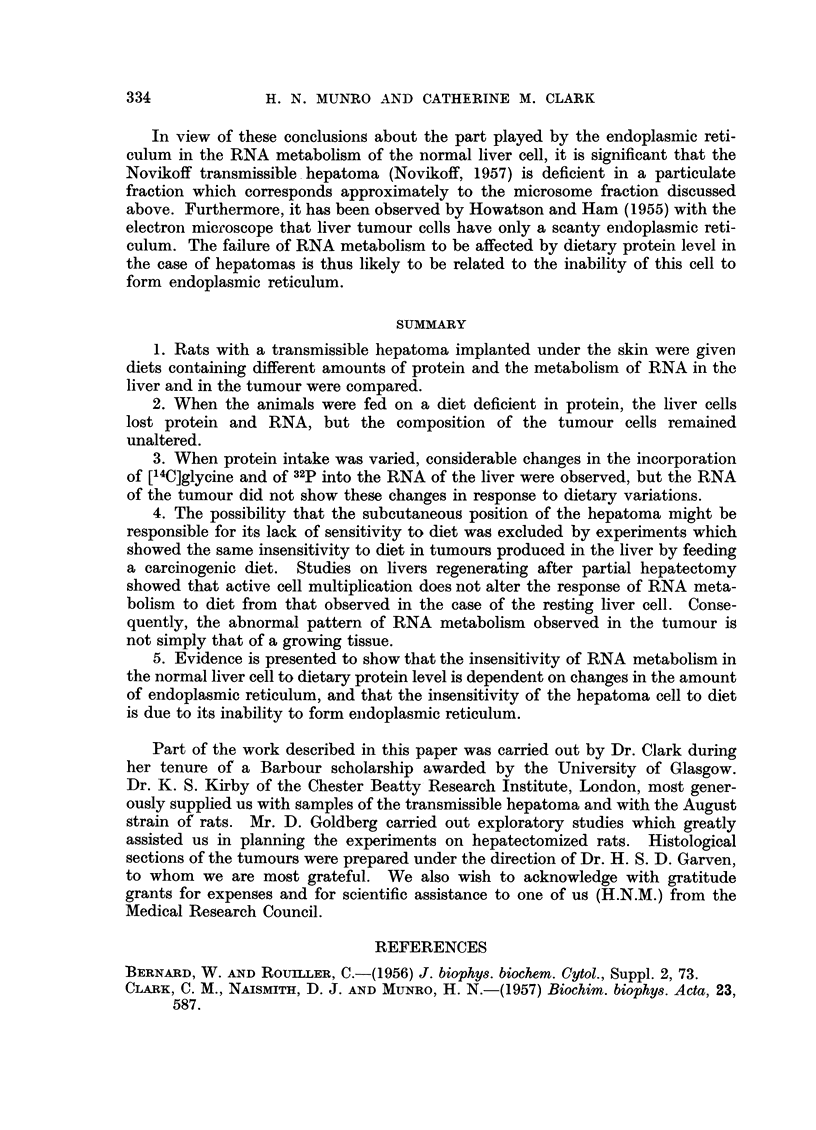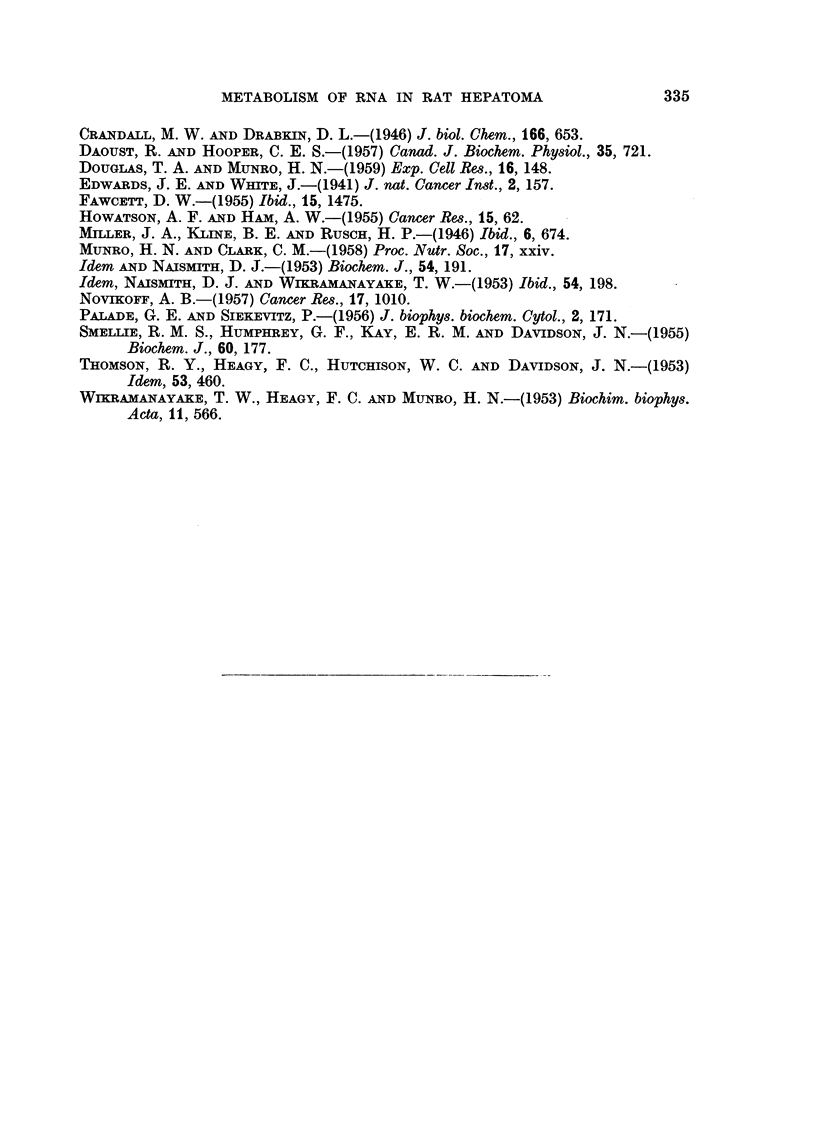# The Influence of Dietary Protein on the Metabolism of Ribonucleic Acid in Rat Hepatoma

**DOI:** 10.1038/bjc.1959.40

**Published:** 1959-06

**Authors:** H. N. Munro, Catherine M. Clark


### THE INFLUENCE OF DIETARY PROTEIN ON THE METABOLISM OF RIBONUCLEIC ACID IN RAT HEPATOMA

H. N. MUNRO AND CATHERINE M. CLARK

*From the Department of Biochemistry, University of Glasgow*

Received for publication March 4, 1959

In a previous study (Clark, Naismith and Munro, 1957) we examined the uptake of [$^{14}$C]glycine and of $^{32}$P by the liver ribonucleic acid (RNA) of normal rats and found conditions under which the incorporation of these precursors was extensively influenced by the protein content of the diet. In particular, rats fasting after a meal containing protein had a much lower uptake of these labelled precursors than animals fasting after a protein-free diet ; uptake was rapidly restored by feeding protein. A considerable degree of sensitivity of liver RNA metabolism to the immediate supply of amino acids was thus established.

It seemed of interest to know whether a tumour derived from liver cells would still exhibit the same fluctuations in RNA metabolism in response to changes in the dietary supply of protein or whether its metabolic activity would be less dependent on dietary conditions. Experiments have accordingly been carried out on rats bearing a transmissible hepatoma implanted subcutaneously, and the responses of the RNA in the tumour and in the normal liver to dietary protein intake have been compared. Whereas the livers of the tumour-bearing animals showed the usual low isotope uptake when the rats were fasted after a meal of protein, the tumour cells did not do so. The hepatoma cells are thus less dependent on dietary conditions than are the parent liver cells.

A preliminary account of these experiments has already appeared (Munro and Clark, 1958).

### EXPERIMENTAL METHODS

### 1. *General techniques*

In the experiments described below, the response of RNA metabolism in liver tumour cells to changes in dietary protein intake has been compared with the response of RNA in normal liver and in regenerating liver.

*Diets and isotope administration.*—Rats of about 200 g. body weight were used in the experiments. For about a week before injection of the isotopes, the animals were fed on synthetic diets (Munro and Naismith, 1953), either containing protein or free from protein. These diets provided about 1200 cal./sq.m. body surface area/day and were given in two portions. The vitamins, minerals and a part of the dietary carbohydrate and fat were fed at 9 a.m. and the rest of the diet (including any protein in the diet) was given at 5 p.m. The animals soon learned to consume these meals rapidly, so that on the morning after the last day of feeding they were all in the post-absorptive state. Rats which had received the protein-free diet and some which had eaten the diet containing protein were then injected intramuscularly at 9 a.m. with $^{32}$P (20 $\mu$c./100 g. body weight) and with [2-$^{14}$C]glycine (10 $\mu$c./100 g. body weight in the experiments on tumour-bearing animals and 5 $\mu$c./100 g. body weight in the studies on liver regeneration). These animals remained fasting until killed 3 or 6 hours later. In addition, some of the rats which had been on the protein-containing diet were fed 2·5 g. of casein solubilized with 0·15 g. $NaHCO_3$, one hour before isotope injection. Thus RNA metabolism could be compared in three groups of animals, (a) those fasting after the protein-free diet, (b) those fasting after the protein-containing diet, and (c) those actively absorbing amino acids from the gut.

*Removal and analysis of tissues.*—Rats from each of the three dietary groups were killed at 3 and at 6 hours after isotope injection. The tissue (liver or tumour) was excised and homogenized in a Nelco blendor in 5 volumes of ice-cold water for 3 minutes and 0·5 volumes of 30 per cent trichloroacetic acid was added. The homogenate was then submitted to procedures described previously (Clark *et al.*, 1957) for quantitative determination of protein N, RNA phosphorus (RNAP) and deoxyribonucleic acid phosphorus (DNAP), and for assessing the specific activity of $^{32}$P in the tissue inorganic phosphate and RNA phosphorus. The specific activities of [$^{14}$C]glycine in the free amino acid pool (trichloroacetic acid-soluble free glycine) and in the glycine portion of the RNA purine bases of the tissue were measured on samples of dinitrophenylglycine prepared from these sources by methods described previously (Clark *et al.*, 1957). These samples were applied evenly to nickel planchets and assayed for radioactivity using an end-window Geiger-Müller counter. The count was prolonged sufficiently to bring the standard error of the count below 5 per cent. A correction for self-absorption by the sample was employed.

In the experiments on liver regeneration, uptake of $^{32}$P by deoxyribonucleic acid (DNA) was also measured in order to be certain that the liver was in a state of active cell multiplication at the time of killing. The procedure used for preparing DNA for assay of its radioactivity was similar to that recommended by Daoust and Hooper (1957). The DNA was separated from the RNA by the Schmidt-Thannhauser procedure as in the case of its quantitative determination, except that the DNA-containing precipitate obtained on acidifying the alkaline digest of nucleic acids was washed three times with 10 per cent trichloroacetic acid before the specific activity of $^{32}$P in the DNA was measured. The low uptake of $^{32}$P by the liver DNA of the control (laparotomized) rats suggests that radioactive contaminants were adequately removed from the DNA by this procedure.

*Method of expressing radioactive uptake.*—The uptakes of $^{32}$P and of [$^{14}$C]-glycine by the various tissue components were first calculated in the form of specific activities (counts/min./100 $\mu$g. P or glycine). The specific activities of [$^{14}$C]glycine samples isolated from the purine bases were then expressed as a percentage of the specific activity of the glycine in the free amino acid pool of the tissue ; similarly, the specific activities of $^{32}$P in the RNA and the DNA were related to that of the tissue inorganic phosphate. These provide relative specific activities which allow for differences in the activities of the precursor pools of different animals. In some experiments, a measure of the total incorporation/cell was also calculated by multiplying the relative specific activity of RNA by the amount of RNAP/mg. DNAP ; this is referred to as the total relative activity.

*Deoxyribonucleic acid content of liver and tumour cell nuclei.*—Nuclei were prepared by the citric acid procedure, as modified by Smellie, Humphrey, Kay and Davidson (1955). In order to obtain clean nuclei, the nuclear fraction was washed with 0·01 M citric acid in the manner described by these authors, the efficacy of washing being controlled microscopically. The nuclei, suspended in 0·01 M citric acid, were counted in a haemacytometer ; a portion of the suspension was also taken for DNA phosphorus determination (Clark *et al.*, 1957). The DNAP content per nucleus was then expressed in pg. (picograms, that is, $10^{-12}$ g.).

*II. Experiments with the transmissible hepatoma*

The main experiments described in this paper were carried out with rats bearing a transmissible hepatoma implanted subcutaneously. The effect of diet on RNA metabolism in the tumour and in the normal liver of the tumour-bearing animals were compared.

Rats of the August strain were inbred to carry the transmissible hepatoma. Dr. K. S. Kirby of the Chester Beatty Institute very kindly supplied us with the transmissible tumour. This originated from an intrahepatic hepatoma produced by feeding 4-fluoro-dimethylaminoazobenzene to rats, and was maintained by subcutaneous inoculation of a suspension of tumour cells. The developed tumour was a solid, fleshy mass. Histologically, the tumour conformed to a Type II hepatoma (Edwards and White, 1941). There were some areas of necrosis in all tumours, but round-cell infiltration and fibrous tissue development were slight.

About 3 weeks before each series of isotope experiments, male rats from the colony were inoculated in the right flank with 1 ml. of a suspension of tumour cells. From this group, animals showing the same degree of tumour development were selected and, for a period of 7 days before injection of the isotopes, some were fed with the protein-containing diet and others with the protein-free diet described above. At the end of this period, the rats were injected with $^{32}P$ and with [2-$^{14}C$]glycine, some animals being in the post-absorptive state, others having been fed casein just prior to injection. The livers and tumours from rats in each of these dietary groups were analyzed as described above.

*III. Experiments with intrahepatic tumours*

In order to be certain that the differences observed between the metabolism of RNA in the transmissible hepatoma and in the normal liver could not be due to the subcutaneous position of the tumour, an experiment was carried out in which tumours were produced in the liver by feeding dimethylaminoazobenzene. Male rats of about 150–180 g. body weight were fed on a carcinogenic diet (Miller, Kline and Rusch, 1946) containing 0·03 per cent dimethylaminoazobenzene for the first month and 0·06 per cent of this carcinogen thereafter. After 6 months liver tumours became apparent and the surviving animals were then fed on the stock diet for a further 2 weeks before commencing the final experimental period.

In this experimental period, some of the tumour-bearing rats were fed for 6 days on the protein-containing diet and others on the protein-free diet. On the morning of the 7th day, they were injected in the fasting state with $^{32}P$ and [2-$^{14}C$]glycine and were killed 6 hours later. In this experiment, none of the animals was fed casein at the time of isotope injection. The tumours were dissected free from the rest of the liver, representative portions of the tumour and of the non-cancerous parts of the liver being taken for measurement of isotope uptake. The tumours and non-cancerous portions of the liver were also examined histologically. The tumours showed some areas of degeneration and neutrophil infiltration ; the areas of liver not transformed into tumour cells exhibited some irregularity of cytoplasmic staining and of nuclear size.

*IV. Experiments with partially hepatectomized rats*

The response of liver RNA metabolism to diet was examined during liver regeneration after partial hepatectomy, in order to determine whether a state of active cell division in the liver alters its sensitivity to diet.

Male albino rats weighing 200–240 g. were fed for 3 days on the protein-containing synthetic diet described above. On the 4th day, half of the animals were transferred to the synthetic protein-free diet while the other rats continued to eat the protein-containing diet. After the second meal of that day had been fully consumed, half the rats in each dietary group were submitted to partial hepatectomy by the method of Crandall and Drabkin (1946). The remaining rats were subjected to laparotomy and served as control animals. Rats in both the experimental and control groups continued to eat the protein-containing or protein-free diets for a further two days. On the morning of the 3rd day after operation, they were injected with $^{32}P$ and with [$^{14}C$]glycine while in the fasting state. Some rats from the hepatectomized and control groups which had received the protein-containing diet were also fed casein just prior to isotope injection. In this way, animals fasting after protein-containing and protein-free diets could be compared with animals actively absorbing amino acids. All animals were killed 6 hours after isotope injection, that is 72 hours after operation. The livers were removed for the measurement of isotope uptake.

### RESULTS

### *Experiments with the transmissible hepatoma*

These studies were carried out on rats bearing a transmissible hepatoma implanted subcutaneously. Comparisons were made of the responses of the tumour and of the normal liver of the same animal to variations in the protein content of the diet.

*Dietary protein and the composition of hepatoma cells and normal liver cells*

In Table I, the protein and RNA content of the hepatoma and of the liver following different diets have been computed per mg. DNAP. The DNA content of the livers of mature animals is a measure of cell number which is unaffected by diet (Thomson, Heagy, Hutchison and Davidson, 1953), and in consequence can be used as a standard of reference in studying changes in other cell constituents. On the protein-deficient diet, the livers of the tumour-bearing rats showed a considerably lower content of protein and of RNA ; this is similar to the response of the livers of normal rats to protein deficiency (Munro, Naismith and Wikramanayake, 1953). In contrast, the amount of protein and of RNA in the hepatoma cell was uninfluenced by the level of protein in the diet.

It is also apparent from Table I that, on each diet, the amount of protein and of RNA/mg. DNAP is considerably less in the case of the hepatoma than in the case of the liver. This comparison of the constituents of the two tissues on the basis of DNA may not be an accurate reflection of differences in cell composition, since some authors have observed that the hepatoma cell nucleus contains less

TABLE I.—*The Influence of Dietary Protein Intake on the Protein and Ribonucleic Acid Content of the Livers and Tumours of Rats Bearing a Transmissible Hepatoma*

The results are the mean data obtained in 6 experiments. Dietary protein had a statistically significant effect on the amounts of protein and RNA in the liver, but no significant action on these constituents of the hepatoma.

| Previous diet | State when killed | Protein N (mg./mg. DNAP) | | RNA P (mg./mg. DNAP) | |
|---|---|---|---|---|---|
| | | Liver | Tumour | Liver | Tumour |
| Protein-free | Post absorptive | 63 | 32 | 4·1 | 1·5 |
| Protein-containing | Post-absorptive | 83 | 32 | 5·3 | 1·4 |
| Protein-containing | After meal of protein | 91 | 32 | 5·3 | 1·6 |

DNA than does the average liver cell nucleus (Thomson *et al.*, 1953). Examination of three specimens of our transmissible hepatoma gave values of 0·61, 0·54 and 0·55 pg. DNAP/nucleus, whereas liver nuclei from the same animals contained 1·01, 1·06 and 1·00 pg./nucleus respectively, figures which are similar to the values for hepatoma and for normal liver nuclei recorded by Thomson *et al.* (1953). In order to use DNA to compare the composition of hepatoma cells with that of liver cells, it would thus be necessary to multiply the amounts of protein and of RNA/mg. DNAP in the liver by a factor of about 1·8, a ratio which is only approximate because of the difficulty of making representative preparations of hepatoma nuclei. Thus, when this difference in the DNA content of the nuclei of the two tissues is taken into account, the hepatoma cells are seen to contain even less protein and RNA in comparison with the normal liver cells than the data in Table I would indicate.

*Uptake of* [$^{14}$C]*glycine by the free amino acids and* $^{32}$P *by the inorganic phosphate of the hepatoma and the liver.*

The specific activities of glycine in the free amino acid pool of the liver and the tumour were examined 3 and 6 hours after isotope injection. Table II shows that the specific activities in the liver and tumour were similar in the case of animals fed on the protein-free diet, but the tumour specific activities were somewhat lower in the two groups of rats previously fed on the diet containing protein. If we assume that the loss of radioactivity from the free amino acid pool of each tissue is exponential over the short period of study, we can calculate the fraction of the activity lost per hour between 3 and 6 hours after injection. The values obtained with the liver and the tumour resemble one another closely (Table II); the values obtained with both tissues are similarly affected by diet.

Uptake of $^{32}$P by the inorganic phosphate pools of the two tissues showed a much wider divergence (Table II). The specific activities in the tumour were about one-third of those in the liver, and the rates of loss of activity between 3 and 6 hours after injection were less in the case of the tumour.

*Diet and the incorporation of precursors into the RNA of the liver and the tumour*

In Table III, the uptakes of [$^{14}$C]glycine and of $^{32}$P by the RNA of the liver and the tumour are expressed in relation to the activities of the precursor pools of glycine and of inorganic phosphate in the tissue (relative specific activities).

TABLE II.—*The influence of Diet on the Specific Activity of* [$^{14}$C]*Glycine in the Free Amino Acid Pool and of* $^{32}$P *in the Tissue Inorganic Phosphate of the Livers and Tumours of Rats Bearing a Transmissible Hepatoma**

| Time after injection (hours) | Previous diet | Fed after isotopes injected | Specific activity of free glycine: Liver | Tumour | Specific activity of inorganic phosphate: Liver | Tumour |
|---|---|---|---|---|---|---|
| 3 | Protein-free | *Nil* | 1828 | 1765 | 5450 | 1460 |
| | Protein-containing | *Nil* | 1605 | 1177 | 5930 | 1825 |
| | Protein-containing | Protein | 1056 | 714 | 4800 | 1710 |
| 6 | Protein-free | *Nil* | 887 | 830 | 3500 | 1295 |
| | Protein-containing | *Nil* | 878 | 669 | 2945 | 1210 |
| | Protein-containing | Protein | 513 | 345 | 2045 | 945 |
| Fraction of activity lost/hour† | Protein-free | *Nil* | 0·241 | 0·252 | 0·148 | 0·040 |
| | Protein-containing | *Nil* | 0·200 | 0·189 | 0·234 | 0·137 |
| | Protein-containing | Protein | 0·242 | 0·244 | 0·285 | 0·198 |

* The data are the mean results obtained in 2 experiments at each time-interval.

† Velocity constant, that is, the difference in $\log_e$ specific activity at the two time-intervals, divided by the time elapsed (3 hours).

The livers of the tumour-bearing animals showed the same sensitivity to dietary protein level which we observed previously in the livers of normal animals (Clark *et al.*, 1957). Liver RNA took up both isotopes extensively when the tumour-bearing animals were fasting after the protein-free diet, whereas there was a reduced uptake of $^{32}$P and almost no uptake of [$^{14}$C]glycine in the case of the rats fasting after the protein-containing diet. Feeding of protein at the time of isotope injection rapidly restored incorporation to a high level. The tumour RNA did not show this sensitivity to dietary protein level. In particular, the low uptake of isotopes observed in the livers of animals fasting after the protein-containing

TABLE III.—*The Influence of Dietary Protein Intake on the Metabolism of Ribonucleic Acid in the Livers and in the Tumours of Rats Bearing a Transmissible Hepatoma**

The data are calculated as relative specific activities

| Time after injection (hours) | Previous diet | Fed after isotope injected | Uptake of [$^{14}$C] glycine†: RNA-adenine: Liver | Tumour | RNA-guanine: Liver | Tumour | Uptake of $^{32}$P‡ by RNA: Liver | Tumour |
|---|---|---|---|---|---|---|---|---|
| 3 | Protein-free | *Nil* | 1·8 | 6·2 | 3·6 | 7·9 | 4·9 | 21·0 |
| | Protein-containing | *Nil* | 0·4 | 10·0 | 0·4 | 12·4 | 2·0 | 18·6 |
| | Protein-containing | Protein | 4·5 | 14·7 | 6·1 | 17·6 | 3·3 | 21·8 |
| 6 | Protein-free | *Nil* | 9·0 | 21·3 | 12·2 | 21·7 | 13·2 | 54·1 |
| | Protein-containing | *Nil* | 1·4 | 30·2 | 0·5 | 28·3 | 6·4 | 50·3 |
| | Protein-containing | Protein | 16·1 | 38·9 | 20·6 | 40·6 | 11·0 | 52·3 |

* Mean data obtained in 2 experiments with [$^{14}$C] glycine and 3 experiments with $^{32}$P.

† The specific activity of the glycine portion of the purine nucleus expressed as a percentage of the specific activity of the free glycine in the tissue.

‡ Specific activity of ribonucleic acid P expressed as a percentage of the specific activity of the inorganic phosphate of the tissue.

diet was not observed in the tumour, and the feeding of protein at the time of isotope injection did not alter $^{32}P$ incorporation into tumour RNA significantly and produced a relatively small change in uptake of [$^{14}C$]glycine.

It is also possible to express the data in the form of total uptake per cell, obtained by multiplying the relative specific activities given in Table III by the amounts of RNAP/mg. DNAP (total relative activities). As might be expected from the comparatively small effect of diet on the amount of RNA in the liver cell and the absence of an effect on tumour cell composition (Table I), these total relative activities follow essentially the same pattern as the relative specific activities given in Table III, and have therefore not been tabulated.

A further difference in the behaviour of RNA metabolism in the liver and in the tumour emerges when the ratio of glycine uptake by guanine relative to glycine uptake by adenine is calculated (Table IV). These ratios have been computed from the data given in Table III and show essentially the same picture at 3 and 6 hours after injection of the isotopes. In the case of liver RNA, the uptake of radioactivity by guanine was greater than the uptake by adenine when the rats were receiving the protein-free diet. This ratio was much lower when the rats were fasting after the protein-containing diet, but rose again when protein was fed at the time of isotope injection. These effects of diet on the relative uptakes of [$^{14}C$]glycine by the guanine and adenine of liver RNA are statistically significant. In contrast, the guanine-adenine ratio of the tumour RNA remains constant on all diets.

TABLE IV.—*The Influence of Dietary Protein Intake on the Relative Uptake of [$^{14}C$]Glycine by the Purine Bases of Ribonucleic Acid in the Livers and in the Tumours of Rats Bearing a Transmissible Hepatoma**

| Time after injection (hours) | Previous diet | Fed after isotope injected | Guanine/adenine ratio for uptake of [$^{14}C$]glycine: Liver | Tumour |
|---|---|---|---|---|
| 3 | Protein-free | *Nil* | 2·1 | 1·3 |
| | Protein-containing | *Nil* | 1·1 | 1·3 |
| | Protein-containing | Protein | 1·5 | 1·3 |
| 6 | Protein-free | *Nil* | 1·5 | 1·1 |
| | Protein-containing | *Nil* | 0·4 | 1·0 |
| | Protein-containing | Protein | 1·3 | 1·1 |

* Mean data obtained in two experiments.

### *Experiments with intrahepatic tumours*

The preceding results show that the response of the transmissible hepatoma to changes in protein intake was different from the response of the liver. In order to establish that these differences between the tumour and the liver were not due to the subcutaneous position of the tumour, a few similar experiments were performed on tumours induced in the liver by feeding dimethylaminoazobenzene to rats. In this case, the response of the tumour and of the non-malignant portions of the liver to diet can be compared under circumstances in which they both receive the same (portal) supply of blood. Fig. 1 shows that, even under these circumstances, the metabolism of RNA in liver tumour cells still differs from that of normal liver cells. Rats with intrahepatic tumours were injected with $^{32}P$ and [$^{14}C$]glycine while fasting after a period of several days feeding on the protein-containing diet or the protein-free diet. In the non-malignant portions of the liver there was the usual *reduced* incorporation of these isotopes when the animals were fasting after a diet containing protein, whereas the RNA of tumours removed from the same livers tended to show a *higher* isotope uptake after the protein-containing diet than after the protein-free diet.

FIG. 1.—Incorporation of $^{32}P$ and of [$^{14}C$]glycine into the RNA of tumours induced in the liver by feeding dimethylaminoazobenzene to rats, compared with their incorporation into the RNA of non-malignant portions of the same livers. Animals fasting after several days of feeding with a protein-free diet are shown by open columns ; animals fasting after a protein-containing diet are shown by solid columns. The data are the mean results obtained in two experiments in which the rats were killed 6 hours after injection of the isotopes. The results are given as relative specific activities, that is, the specific activity of RNA phosphorus expressed as a percentage of the specific activity of the inorganic phosphate of the tissue, and the specific activity of the glycine portions of RNA adenine and guanine expressed as a percentage of the specific activity of free glycine.

### *Experiments with partially hepatectomized rats*

The sensitivity of the liver to protein level in the diet was studied during regeneration after partial hepatectomy, in order to determine whether the change in RNA metabolism observed in the hepatoma could result from its being in a state of active cell division. Rats were fed on the protein-containing or protein-free diets for the first 72 hours after hepatectomy or laparotomy (control procedure), and then uptake of $^{32}P$ and of [$^{14}C$]glycine by the liver RNA was measured. The results (Table V) are expressed in the form of relative specific activities ; since the amounts of RNAP/mg. DNAP were not greatly affected by diet, calculation of the data in the form of total isotope uptake/cell (total relative activities) would give essentially the same picture. In both the hepatectomized and the laparotomized series of animals, incorporation was affected in a similar manner by diet; there was a marked reduction in uptake of both isotopes into RNA when the rats were fasting after the diet containing protein and a restoration of uptakc when protein was fed at the time of isotope injection (Table V). Uptake of $^{32}P$ into liver DNA was considerably elevated in the hepatectomized group, a finding which is consistent with a state of active cell division. It can thus be concluded that an increase in the rate of cell multiplication does not by itself reduce the sensitivity of liver RNA metabolism to dietary protein level.

TABLE V.—*The Influence of Dietary Protein Intake on the Metabolism of Ribonucleic Acid in the Liver During Regeneration After Partial Hepatectomy, Compared with Laparotomy**

| Operation | Previous diet | Fed after isotope injection | Mg. RNAP per mg. DNAP | Uptake of [$^{14}C$] glycine† by RNA: Adenine | Uptake of [$^{14}C$] glycine† by RNA: Guanine | Uptake of $^{32}P$‡: RNA | Uptake of $^{32}P$‡: DNA |
|---|---|---|---|---|---|---|---|
| Hepatectomy | Protein-free | *Nil* | 3·8 | 7·9 | 18·4 | 15·1 | 4·9 |
| | Protein-containing | *Nil* | 3·6 | 0·8 | 1·0 | 5·9 | 2·3 |
| | Protein-containing | Protein | 3·8 | 12·1 | 18·8 | 14·7 | 3·4 |
| Laparotomy | Protein-free | *Nil* | 3·1 | 4·7 | 6·7 | 9·8 | 0·9 |
| | Protein-containing | *Nil* | 3·5 | 0·3 | 0·2 | 4·8 | 0·8 |
| | Protein-containing | Protein | 3·4 | 7·2 | 11·2 | 8·8 | 0·8 |

* The data are the mean results obtained in two experiments, in which the rats were killed 6 hours after injection of the isotopes.

† The specific activity of the glycine portion of the purine nucleus expressed as a percentage of the specific activity of free glycine in the tissue (relative specific activity).

‡ The specific activity of ribonucleic acid or deoxyribonucleic acid P expressed as a percentage of the specific activity of the inorganic phosphate of the tissue (relative specific activity).

### DISCUSSION

The response of liver RNA metabolism to changes in protein intake has been described in a previous paper (Clark *et al.*, 1957). Compared with rats fasting after receiving a protein-free diet, animals fasting after a diet containing protein were observed to incorporate less $^{32}P$ and very much less [$^{14}C$]glycine into liver RNA. Incorporation levels in these animals were dramatically raised when protein was fed at the time of isotope injection. A considerable degree of sensitivity of liver RNA metabolism to the available supply of amino acids was thus established.

In the present series of experiments, we have investigated the question of whether a tumour derived from liver cells would still exhibit fluctuations in RNA metabolism with changes in protein intake or whether its metabolic activities would be less dependent on dietary conditions. For our main series of experiments a transmissible hepatoma implanted subcutaneously was preferred to the use of intrahepatic tumours produced by feeding carcinogenic azo-dyes, because the type of tumour produced in the latter way cannot be controlled (Edwards and White, 1941), and furthermore the distinction between malignant and non-malignant parts of the liver sometimes presents difficulties. When the protein intake of rats bearing the transmissible hepatoma was varied, liver RNA showed the same sensitivity to diet observed previously in the case of normal rats, but the tumour did not show this response. The protein and RNA contents of the tumour cell were unaffected by the level of protein intake (Table I) and the uptake of isotopes by the RNA of the tumour (Tables III and IV) also did not respond in the characteristic manner of liver cells.

Certain possible reasons for these differences between the tumour and the liver can be excluded. In the first place, the tumour was supplied by the systemic circulation whereas the liver received portal blood, and this might be the reason why the liver alone was affected by the supply of amino acids coming from the diet. However, this explanation was eliminated by a few experiments carried out on hepatomas which had been induced within the liver by feeding dimethylaminoazobenzene. These intrahepatic tumours were also found to be insensitive to dietary changes which caused the usual alterations in RNA metabolism in adjacent non-malignant areas of the same livers (Fig. 1). A second possibility is that the tumour was insensitive to diet because it was tissue in a state of active cell division. A study of the effect of dietary protein level on RNA metabolism in regenerating liver (Table V) showed, however, that stimulation of cell division in the liver does not by itself affect the capacity of the liver cell to respond to the protein content of the diet. Consequently, the peculiarities of RNA metabolism in the hepatoma are not simply the result of rapid cell division.

The evidence thus points to a fundamental difference between the metabolism of RNA in the hepatoma cell and in the normal liver cell, RNA metabolism in the hepatoma having lost its capacity to respond to changes in dietary protein level which affect RNA metabolism in normal hepatocytes. In order to offer an explanation of the insensitivity of the tumour cell to diet, it is necessary to consider first the mechanism by which the RNA of normal liver cells responds to the protein content of the diet. In our previous study (Clark *et al.*, 1957) we reached the conclusion that the supply of amino acids coming to the liver influenced the rate of RNA breakdown. The striking effect which protein intake has on the uptake of isotopes by liver RNA was attributed by us to differences in the extent to which RNA breakdown products dilute isotopic labelling in the pools of RNA precursor compounds. Some indication has been obtained by cell fractionation procedures (Wikramanayake, Heagy and Munro, 1953) of the intracellular location of the RNA which is affected in this way by dietary protein level. Rats receiving a protein-free diet showed a loss of RNA from the liver which was essentially confined to the microsomal fraction of the cell precipitated by centrifuging at a comparatively low speed (18,000 *g.* with 0·25 M. sucrose homogenates). The sensitivity of RNA metabolism to dietary protein level thus appears to be associated with changes in this cell fraction. Microsome fractions sedimented from liver and other tissues at these relatively low speeds contain RNA in the form of ribonucleoprotein particles attached to small vesicles (Palade and Siekevitz, 1956 ; Douglas and Munro, 1959) which are derived from the endoplasmic reticulum of the intact cell (Palade and Siekevitz, 1956). Loss of RNA from this fraction should therefore be represented in the intact liver cell by changes in the endoplasmic reticulum. Two independent studies of liver cells with the electron microscope (Fawcett, 1955 ; Bernard and Rouiller, 1956) have in fact shown that the endoplasmic reticulum is greatly reduced or even absent after several days of fasting and reappears within a few hours of giving a meal rich in protein. A diet low in protein is much less effective in causing restoration of the reticulum (Fawcett, 1955). It can be concluded that these losses and gains in endoplasmic reticulum, with its adherent ribonucleoprotein particles, account for the sensitivity of RNA metabolism in the liver to changes in protein intake.

In view of these conclusions about the part played by the endoplasmic reticulum in the RNA metabolism of the normal liver cell, it is significant that the Novikoff transmissible hepatoma (Novikoff, 1957) is deficient in a particulate fraction which corresponds approximately to the microsome fraction discussed above. Furthermore, it has been observed by Howatson and Ham (1955) with the electron microscope that liver tumour cells have only a scanty endoplasmic reticulum. The failure of RNA metabolism to be affected by dietary protein level in the case of hepatomas is thus likely to be related to the inability of this cell to form endoplasmic reticulum.

### SUMMARY

1. Rats with a transmissible hepatoma implanted under the skin were given diets containing different amounts of protein and the metabolism of RNA in the liver and in the tumour were compared.

2. When the animals were fed on a diet deficient in protein, the liver cells lost protein and RNA, but the composition of the tumour cells remained unaltered.

3. When protein intake was varied, considerable changes in the incorporation of [$^{14}$C]glycine and of $^{32}$P into the RNA of the liver were observed, but the RNA of the tumour did not show these changes in response to dietary variations.

4. The possibility that the subcutaneous position of the hepatoma might be responsible for its lack of sensitivity to diet was excluded by experiments which showed the same insensitivity to diet in tumours produced in the liver by feeding a carcinogenic diet. Studies on livers regenerating after partial hepatectomy showed that active cell multiplication does not alter the response of RNA metabolism to diet from that observed in the case of the resting liver cell. Consequently, the abnormal pattern of RNA metabolism observed in the tumour is not simply that of a growing tissue.

5. Evidence is presented to show that the insensitivity of RNA metabolism in the normal liver cell to dietary protein level is dependent on changes in the amount of endoplasmic reticulum, and that the insensitivity of the hepatoma cell to diet is due to its inability to form endoplasmic reticulum.

Part of the work described in this paper was carried out by Dr. Clark during her tenure of a Barbour scholarship awarded by the University of Glasgow. Dr. K. S. Kirby of the Chester Beatty Research Institute, London, most generously supplied us with samples of the transmissible hepatoma and with the August strain of rats. Mr. D. Goldberg carried out exploratory studies which greatly assisted us in planning the experiments on hepatectomized rats. Histological sections of the tumours were prepared under the direction of Dr. H. S. D. Garven, to whom we are most grateful. We also wish to acknowledge with gratitude grants for expenses and for scientific assistance to one of us (H.N.M.) from the Medical Research Council.

### REFERENCES

Bernard, W. and Rouiller, C.—(1956) *J. biophys. biochem. Cytol.*, Suppl. 2, 73.

Clark, C. M., Naismith, D. J. and Munro, H. N.—(1957) *Biochim. biophys. Acta*, **23**, 587.

CRANDALL, M. W. AND DRABKIN, D. L.—(1946) *J. biol. Chem.*, **166**, 653.
DAOUST, R. AND HOOPER, C. E. S.—(1957) *Canad. J. Biochem. Physiol.*, **35**, 721.
DOUGLAS, T. A. AND MUNRO, H. N.—(1959) *Exp. Cell Res.*, **16**, 148.
EDWARDS, J. E. AND WHITE, J.—(1941) *J. nat. Cancer Inst.*, **2**, 157.
FAWCETT, D. W.—(1955) *Ibid.*, **15**, 1475.
HOWATSON, A. F. AND HAM, A. W.—(1955) *Cancer Res.*, **15**, 62.
MILLER, J. A., KLINE, B. E. AND RUSCH, H. P.—(1946) *Ibid.*, **6**, 674.
MUNRO, H. N. AND CLARK, C. M.—(1958) *Proc. Nutr. Soc.*, **17**, xxiv.
*Idem* AND NAISMITH, D. J.—(1953) *Biochem. J.*, **54**, 191.
*Idem*, NAISMITH, D. J. AND WIKRAMANAYAKE, T. W.—(1953) *Ibid.*, **54**, 198.
NOVIKOFF, A. B.—(1957) *Cancer Res.*, **17**, 1010.
PALADE, G. E. AND SIEKEVITZ, P.—(1956) *J. biophys. biochem. Cytol.*, **2**, 171.
SMELLIE, R. M. S., HUMPHREY, G. F., KAY, E. R. M. AND DAVIDSON, J. N.—(1955) *Biochem. J.*, **60**, 177.
THOMSON, R. Y., HEAGY, F. C., HUTCHISON, W. C. AND DAVIDSON, J. N.—(1953) *Idem*, **53**, 460.
WIKRAMANAYAKE, T. W., HEAGY, F. C. AND MUNRO, H. N.—(1953) *Biochim. biophys. Acta*, **11**, 566.